# Cortical Iron and Mesostriatal Dopamine Function in Schizophrenia: A Positron Emission Tomography and Magnetic Resonance Imaging Study

**DOI:** 10.1093/schbul/sbag045

**Published:** 2026-07-23

**Authors:** Luke James Vano, Jan Sedlacik, Stephen John Kaar, Grazia Rutigliano, Richard Carr, Alaine Berry, Ben Statton, Amir Fazlollahi, Mattia Veronese, Oliver David Howes, Robert Ali McCutcheon

**Affiliations:** Department of Psychosis Studies, Institute of Psychiatry, Psychology & Neuroscience, King’s College London, London, SE5 8AF, United Kingdom; Psychiatric Imaging Group, MRC Laboratory of Medical Sciences, Hammersmith Hospital, London, W12 0HS, United Kingdom; Institute of Clinical Sciences, Faculty of Medicine, Imperial College London, London, SW7 5NH, United Kingdom; South London and Maudsley NHS Foundation Trust, London, SE5 8AZ, United Kingdom; Psychiatric Imaging Group, MRC Laboratory of Medical Sciences, Hammersmith Hospital, London, W12 0HS, United Kingdom; Institute of Clinical Sciences, Faculty of Medicine, Imperial College London, London, SW7 5NH, United Kingdom; Mansfield Centre for Innovation - MR Facility, MRC Laboratory of Medical Sciences, Hammersmith Hospital, London, W12 0HS, United Kingdom; Department of Psychosis Studies, Institute of Psychiatry, Psychology & Neuroscience, King’s College London, London, SE5 8AF, United Kingdom; Psychiatric Imaging Group, MRC Laboratory of Medical Sciences, Hammersmith Hospital, London, W12 0HS, United Kingdom; Institute of Clinical Sciences, Faculty of Medicine, Imperial College London, London, SW7 5NH, United Kingdom; Division of Psychology and Mental Health, Faculty of Biology, Medicine, and Health, University of Manchester, Manchester, M13 9PL, United Kingdom; Department of Psychosis Studies, Institute of Psychiatry, Psychology & Neuroscience, King’s College London, London, SE5 8AF, United Kingdom; Psychiatric Imaging Group, MRC Laboratory of Medical Sciences, Hammersmith Hospital, London, W12 0HS, United Kingdom; Institute of Clinical Sciences, Faculty of Medicine, Imperial College London, London, SW7 5NH, United Kingdom; Department of Psychosis Studies, Institute of Psychiatry, Psychology & Neuroscience, King’s College London, London, SE5 8AF, United Kingdom; Psychiatric Imaging Group, MRC Laboratory of Medical Sciences, Hammersmith Hospital, London, W12 0HS, United Kingdom; Institute of Clinical Sciences, Faculty of Medicine, Imperial College London, London, SW7 5NH, United Kingdom; Mansfield Centre for Innovation - MR Facility, MRC Laboratory of Medical Sciences, Hammersmith Hospital, London, W12 0HS, United Kingdom; Psychiatric Imaging Group, MRC Laboratory of Medical Sciences, Hammersmith Hospital, London, W12 0HS, United Kingdom; Institute of Clinical Sciences, Faculty of Medicine, Imperial College London, London, SW7 5NH, United Kingdom; Mansfield Centre for Innovation - MR Facility, MRC Laboratory of Medical Sciences, Hammersmith Hospital, London, W12 0HS, United Kingdom; Department of Radiology, Royal Melbourne Hospital, University of Melbourne, Parkville, VIC, 3050, Australia; Faculty of Health, Medicine and Behavioural Sciences, Queensland Brain Institute, The University of Queensland, St Lucia, QLD, 4067, Australia; Department of Neuroimaging, King's College London, London, SE5 8AF, United Kingdom; Department of Information Engineering, University of Padua, 35131, Padova, PD, Italy; Department of Psychosis Studies, Institute of Psychiatry, Psychology & Neuroscience, King’s College London, London, SE5 8AF, United Kingdom; Institute of Clinical Sciences, Faculty of Medicine, Imperial College London, London, SW7 5NH, United Kingdom; Department of Psychosis Studies, Institute of Psychiatry, Psychology & Neuroscience, King’s College London, London, SE5 8AF, United Kingdom; Department of Psychiatry, University of Oxford, Oxford, OX3 7JX, United Kingdom; Oxford Health NHS Foundation Trust, Warneford Hospital, Oxford, OX3 7JX, United Kingdom

**Keywords:** cortex, iron, basal ganglia, dopamine, neuromelanin

## Abstract

**Background and Hypothesis:**

Elevated postmortem iron in Brodmann areas 10-11 has recently been linked to schizophrenia. Although in vivo studies have linked subcortical iron abnormalities to disease-related striatal hyperdopaminergia*,* in vivo cortical iron alterations have not been previously examined. We therefore used neuroimaging to test whether cortical iron is elevated in individuals with schizophrenia and whether this correlated with mesostriatal dopamine function.

**Study Design:**

We acquired quantitative susceptibility mapping magnetic resonance imaging (MRI) to measure magnetic susceptibility (χ), a marker of iron, in 149 participants aged 18-45 (73 with schizophrenia and 76 matched healthy controls). Subsets of patients with schizophrenia underwent [18F]-DOPA PET to estimate striatal dopamine synthesis capacity (*n* = 39) and neuromelanin-sensitive MRI of the dopaminergic midbrain (*n* = 68), as neuromelanin is a byproduct of dopamine synthesis.

**Study Results:**

Primary analyses showed no significant case–control differences in χ in the whole cortex (*P* = .675) or Brodmann areas 10-11 (*P* = .537). Exploratory analyses examined χ for 360 cortical regions, correcting for multiple comparisons. Two left temporo-parieto-occipital junction regions showed significantly elevated χ in schizophrenia: the posterior temporo-parieto-occipital junction the posterior temporo-parieto-occipital junction (*d* = 0.752, *P* < .001) and the superior temporal visual area (*d* = 0.638, *P* = .034). Mean χ across these regions inversely correlated with dopamine synthesis capacity in the associative (*r* = –0.37, *P* = .048) and limbic (*r* = –0.34, *P* = .048) striatum, and with neuromelanin-sensitive MRI values in the dopaminergic midbrain (*r* = –0.35, *P* = .005), corrected for multiple comparisons.

**Conclusions:**

This study provides the first in vivo evidence of elevated cortical iron in schizophrenia and links this to mesostriatal dopamine dysfunction.

## Introduction

While cortical thinning^1^^,^^2^ and altered functional connectivity^3^ are established features of schizophrenia, their underlying biological origin remains unresolved. Iron dysregulation has been proposed as a contributing factor,^4^ given its essential role for many neurophysiological processes including myelination, neurotransmitter synthesis, and neurogenesis.^5^ Iron levels must be tightly regulated, as excess brain iron can damage grey matter through increased oxidative stress, lipid peroxidation, and neuroinflammation.^5^ A recent postmortem study of Brodmann areas (BA) 10-11 reported elevated iron but reduced ferritin—the major storage protein of iron—in schizophrenia.^4^ Because ferritin normally sequesters iron in a redox-inactive form, it was hypothesized that this may indicate an increased pool of chemically active iron, leading to the cortical thinning observed on structural magnetic resonance imaging (MRI) in schizophrenia.^1^^,^^2^

Quantitative susceptibility mapping (QSM) is an MRI technique that estimates tissue magnetic susceptibility (χ), which is increased by iron.^6^ Previous case–control studies have only examined the subcortex, linking early-course schizophrenia to lower subcortical χ,^7–10^ consistent with iron loss. The lack of previous studies conducting QSM cortical examination makes it unclear whether cortical χ is higher or lower in patients with schizophrenia and if this is associated with cortical thinning or psychotic symptoms.

We recently showed in patients with schizophrenia that χ in the substantia nigra and ventral tegmental area (SN-VTA) inversely correlated with striatal dopamine synthesis capacity, assessed using positron emission tomography (PET) to measure [18F]-DOPA uptake (K_i_^cer^).^9^ This suggests that low SN-VTA iron levels may contribute to elevated striatal dopamine function in schizophrenia, consistent with preclinical evidence linking iron deficiency with striatal hyperdopaminergia.^11–14^ Cortical glutamatergic projections regulate dopamine function in the SN-VTA and striatum. Given that iron modulates glutamatergic neurotransmission^15^ and may contribute to cortical dysfunction,^4^ cortical iron abnormalities may also alter mesostriatal dopamine function. SN-VTA dopamine production may be indexed by neuromelanin-sensitive MRI (NM-MRI), as neuromelanin is a byproduct of dopamine metabolism.^16^

In this study, we performed cortical QSM in a case–control cohort for the first time to investigate whether cortical iron alterations are present in schizophrenia. We hypothesized that mean χ would be greater in patients with early-course schizophrenia across the whole cortex and within BA 10-11. We additionally conducted an exploratory analysis across cortical subregions. For regions showing significant cortical χ differences, we examined associations with clinical symptom scores, striatal K_i_^cer^, and SN-VTA NM-MRI within the schizophrenia group to test whether cortical iron alterations were related to psychotic symptoms and mesostriatal dopamine function.

## Methods

### Participants

Ethical approval was granted by the London-Dulwich Research Ethics Committee for healthy participants and by the Office for Research Ethics Committees Northern Ireland for individuals with schizophrenia (NCT04038957). All participants provided written informed consent. Recruitment was limited to individuals aged 18-45 years, to target those in the early stages of illness.

Individuals diagnosed with schizophrenia were referred from community mental health services in London, UK. Diagnoses were confirmed by a study psychiatrist using clinical records and the Structured Clinical Interview for DSM-5 (SCID-5).^17^ Participants were excluded if they had comorbid psychiatric diagnoses, current or past substance use disorders (excluding nicotine), were taking non-antipsychotic psychotropic medications, more than one antipsychotic, or current or prior clozapine treatment. For those undergoing PET, their history of radiation exposure could not exceed 6 mSv in the previous 12 months.

Healthy control participants were recruited through public advertisements in London, UK. A psychiatrist screened volunteers, excluding those with personal psychiatric history, psychotropic use, substance use disorder (excluding nicotine), or a first-degree relative with schizophrenia.

All participants underwent a urine drug screen (UDS) prior to their MRI. Individuals testing positive for stimulants, benzodiazepines, or opiates were excluded. Participants who were positive for delta-9-tetrahydrocannabinol (THC), which may persist in urine for extended periods,^18^ were able to participate as long as they had not used it in the past month. Smoking status was recorded as current, former, or never.

### Clinical Assessments

Demographic and clinical data were collected from all participants. Patients completed the Positive and Negative Syndrome Scale (PANSS),^19^ the Brief Negative Symptom Scale (BNSS),^20^ and the Clinical Global Impression-Severity scale (CGI-S).^21^ Patient antipsychotic doses were converted to chlorpromazine daily equivalents.^22^

### MRI Acquisition

We scanned participants using a 3 T MRI scanner (Magnetom Prisma, Siemens Healthineers, Erlangen, Germany) with a 64-channel receive head coil. Longitudinal relaxation time weighted (T1w) images were generated with a magnetization prepared rapid gradient echo (MPRAGE; acquisition time (TA) = 5 min 35 s, repetition time (TR) = 2300 ms, inversion time (TI) = 900 ms, echo time (TE) = 2.91 ms, flip angle = 9°, 176 slices with the voxel size = 1 × 1 × 1mm^3^). QSM maps were calculated from magnitude and phase images generated from a 3D gradient recall echo (GRE) sequence (TA = 8 min 5 s, TR = 50 ms, first TE at 5.84 ms with 8 subsequent echoes 4.79 ms apart, flip angle = 15°, 144 slices with the voxel size = 1 × 1 × 1mm^3^). χ is reported in parts per billion. NM-MRI was acquired using an axial 2D gradient echo sequence with a magnetization transfer pulse (TA = 5 min 50 s, TR = 359 ms, TE = 2.85 ms, flip angle = 40°, 12 slices with the voxel size = 0.8 × 0.8 × 2 mm^3^). QSM and NM-MRI images were quality controlled by investigators LJV and JS, who were blinded to diagnosis and used best practice guidelines.^23^^,^^24^

### QSM Preprocessing

QSM maps can be distorted near B0 inhomogeneities, such as air-tissue or bone-tissue boundaries next to the orbitalfrontal cortex and inferior temporal lobes. These areas exhibit signal drop-out on GRE magnitude images and are typically excluded by conservative brain masking. Therefore, we generated a brain mask from the first-echo magnitude image using the FMRIB Software Laboratory (FSL) Brain Extraction Tool, which we then eroded by 5%.^25^ As this mask lacks precision at cortical boundaries, FreeSurfer was used to generate a Desikan-Killiany-Tourville cortical atlas on T1w images.^26^ The GRE magnitude image was co-registered to the T1w image using NiftyReg with a 15 mm spline grid to restrict non-linear warping to regions with substantial distortion.^27^ The resulting transformations were used to map the FreeSurfer mask into GRE space. QSM processing was limited to voxels within both masks. QSM maps were referenced to the mean χ of all voxels within the final whole brain mask.

Frequency shift was computed from brain-masked phase images acquired with the GRE sequence, using Fit_ppm_complex.m from the Morphology Enabled Dipole Inversion (MEDI) toolbox.^28^ Local frequency was estimated via projection onto dipole fields,^29^ and QSM maps were generated using iterative Tikhonov dipole inversion.^30^ Only cortical voxels with Desikan-Killiany-Tourville labels >1000, as well as hippocampal voxels^17^^,^^31^, were retained. Figure S1 displays the QSM images for a representative control and patient.

For our region of interest (ROI) analysis, we employed the extended Human Connectome Project parcellation (HCPex v1.1).^32^ As this atlas is in the Montreal Neuroimaging Institute (MNI) space, we used sMRIprep (v0.17.0) to normalize the T1w images.^33^ The relevant inverse affine transformation-matrices and warp-fields were applied to move the atlas from the MNI to GRE space. FreeSurfer cortical voxels were re-labeled based on overlapping HCPex atlas voxels or nearest-neighbor assignment. ROIs corresponding to Brodmann areas 10-11 (labels 134, 135, 150, 151, 156, 158, 314, 315, 316, 330, 336, 338, 148, 152, 157, 328, 332, and 337) were combined into a single mask for our primary analysis. We then applied the mask generated from the first-echo magnitude image, which removes voxels experiencing significant distortion, to generate our final cortical parcellated atlas. The number of voxels for each ROI in this mask was divided by the value from the original cortical parcellation to estimate the effect that distortion had on an ROI.

Any HCPex labels showing significant case–control χ differences were moved from MNI space to T1w space using the relevant inverse affine transformation-matrices and warp-fields. Cortical thickness within these labels was then quantified using the standard FreeSurfer recon-all pipeline.^34^ At each vertex, cortical thickness was computed as the distance between the corresponding vertices of the white matter and pial surfaces.^35^ Mean χ values were additionally extracted for the following HCPex subcortical ROIs: striatum (combining the nucleus accumbens, putamen, and caudate nucleus masks), globus pallidus, and thalamus. SN-VTA χ was extracted from the same mask used for the NM-MRI analysis.

For the voxelwise analysis, cortical QSM maps were transformed into MNI space using the relevant affine and nonlinear transformations. To minimize the influence of outliers, voxels with χ values below the 1st percentile or above the 99th percentile were excluded. Voxelwise analysis was restricted to the remaining cortical voxels, which were smoothed with a Gaussian kernel (σ = 3 mm). Figure S2 shows the voxelwise mean of all QSM images passing quality control in MNI space.

### NM-MRI Preprocessing

We followed our previously described NM-MRI automated analysis pipeline (accessible from: https://github.com/lukevano/KCL_Neuromelanin-MRI).^36^ Participant NM-MRI images were co-registered to T1-weighted images to allow a midbrain atlas^9^ containing SN-VTA and crus cerebri masks to be moved from the MNI to the original NM-MRI space. NM-MRI maps were converted to NM-MRI contrast-to-noise ratio (NM-CNR) images using the following voxelwise calculation:

(voxel intensity − mode crus cerebri voxel intensity)/mode crus cerebri voxel intensity.

The mode crus cerebri intensity was estimated by applying a kernel-smoothing-function to the distribution of voxels within the crus cerebri mask using the gaussian_kde function from the Python package SciPy v1.12.0.^37^

### PET Acquisition

All patients with schizophrenia who underwent the MRI scan were also offered a PET scan. The scan protocol and analysis pipeline followed our standard procedures.^38^^,^^39^ A SIGNA General Electric 3 T PET/MR scanner was used. Approximately one hour prior to the PET scan, participants orally ingested carbidopa (150 mg) and entacapone (400 mg) to increase the signal-to-noise ratio of the PET data.^40^ Immediately before the PET sequence initiation, a zero-time echo MRI scan was completed for attenuation correction. An MR localizer was completed to define the PET field of view (including the entire brain and centered on the thalamus). A maximum activity of 150 MBq [18F]-DOPA was injected. This was diluted to 10 mL with 0.9% normal saline and given as a bolus over 20 s, followed by a further 10 mL saline flush.

PET emission data were collected over 95 min. The list-mode data were temporally segmented into 26 frames comprising an initial 30-s background frame, followed by four 60-s frames, three 120-s frames, three 180-s frames, and fifteen 300-s frames. Image reconstruction was performed using a 3D reprojection algorithm.^41^

### PET Image Processing

For motion correction, individual frames were realigned to the frame acquired at 15 min post-injection, in line with established procedures.^42^ Motion-corrected frames were subsequently summed to generate a mean dynamic image for each participant. Attenuation correction was applied using the zero-time echo MRI sequence. Each participant’s summed PET image was then rigidly co-registered to their corresponding T1-weighted image. An atlas containing the striatal subdivisions (limbic, associative, and sensorimotor) and cerebellum^43^ was moved from MNI to each participant’s PET space.

Prior to statistical analysis, all datasets underwent quality control to verify adequate motion correction, accurate region segmentation, and physiologically plausible parameter estimates, excluding values that were negative or outside expected biological ranges, following previously described criteria.^42^

Dopamine synthesis capacity was quantified by estimating the influx rate constant (K_i_^cer^) for [18F]-DOPA in striatal subdivisions, using the cerebellum as a reference tissue. K_i_^cer^ values were derived with Patlak–Gjedde graphical analysis,^41^ implemented via a custom MATLAB-based pipeline incorporating Piwave,^44^ the FDOPA package, and Statistical Parametric Mapping (SPM 12).^45^

## Statistical Analysis

### Baseline Clinicodemographic Analysis

We examined for case–control clinicodemographic differences with chi-squared tests for categorical variables and independent sample *t*-tests for continuous variables.

### Case–Control ROI QSM Analysis

Participants were included in the ROI analysis if ≥70% of voxels within the region were retained after masking. This threshold was chosen for consistency with previous cortical analyses using our QSM preprocessing pipeline.^46^ Results were only reported if at least 50 participants remained in each group for an ROI.

Our 2 predefined primary analyses compared mean χ between patients and controls in (1) the whole cortex and (2) the combined BA 10-11 ROI. We used independent sample *t*-tests to assess the significance and Cohen’s d for the effect size.

For our exploratory analysis, we tested χ case–control differences independently across all HCPex cortical ROIs (360 regions). Significance was assessed via false discovery rate (FDR) correction using the Benjamini-Hochberg method (*P* < .05). If χ case–control differences were identified for any ROIs, the mean χ across these ROIs was calculated. Then a robust linear regression model was used to determine whether these case–control differences in χ were influenced by clinical confounders (age, sex, current or past smoking history, THC-positive UDS, and mean cortical thickness within these ROIs).

### Correlating QSM in Significant ROIs with Clinical and Other Neuroimaging Measures

In the patient group, mean χ for the significant ROIs were correlated with PANSS positive, negative, and general scores, significant ROI thickness, mean K_i_^cer^ in the striatal subregions, mean χ in the subcortical subregions, and mean SN-VTA NM-CNR. An FDR correction was applied within each modality.

### Case–Control Voxelwise QSM Analysis

We performed a voxelwise analysis across the entire cortex to identify clusters showing significant case–control differences. We used threshold-free cluster enhancement with family-wise error (FWE) correction at 10 000 permutations (*P* < .05) with the function “randomise” in FSL.^47^

## Results

### Baseline Clinicodemographic Analysis

QSM maps were generated for 171 participants (86 healthy controls and 85 with early course schizophrenia). Twenty-two of these maps failed quality control: 7 for excessive movement (3 healthy controls and 4 with schizophrenia) and 15 for artifacts (7 healthy controls and 8 with schizophrenia). The final QSM dataset included 76 healthy controls and 73 with schizophrenia (17 were antipsychotic-free). In the schizophrenia group, 68 had successfully analyzed NM-MRI data (3 removed for excessive movement) and 39 PET data (2 removed for excessive movement). Clinicodemographic information is reported in Table 1. There were no significant case–control differences in age, sex, ethnicity, or THC-positive status but current smoking was more prevalent in the schizophrenia group compared to controls (34% vs. 16%; *P* = .03).

**Table 1:** Participant Clinicodemographic Data of Those with Usable Quantitative Susceptibility Mapping Data

	Healthy controls	Schizophrenia	Test statistic
	(*n* = 76)	(*n* = 73)	
Male	53 (70%)	52 (71%)	χ^2^_1_ = 0; *P* = .98
Age	32.1 (0.73)	31.8 (0.82)	t = -0.88; *P* = .38
**Ethnicity**			
Asian	10 (13%)	10 (14%)	χ^2^_3_ = 1.72; *P* = .63
Black	32 (42%)	38 (52%)	
White	29 (38%)	22 (30%)	
Mixed	5 (7%)	3 (4%)	
**Smoking status**			
Current smoker	12 (16%)	25 (34%)	χ^2^_2_ = 6.84; *P* = .03
Past Smoker	13 (17%)	9 (12%)	
Never Smoked	51 (67%)	39 (54%)	
THC-positive UDS	18 (24%)	18 (25%)	χ^2^_1_ = 0.02; *P* = .89
**Neuroimaging measures**			
Number with NM-MRI data	Not analyzed for healthy controls	68 (93%)	
Number with PET data		39 (53%)	
**Clinical measures**			
PANSS Total		74.5 (1.9) [36-100]	
PANSS Positive		17.4 (0.8) [7-31]	
PANSS Negative		20.7 (0.7) [8-38]	
PANSS General	Not performed for Healthy Controls	36.3 (0.9) [16-49]	
BNSS		31.5 (2) [1-65]	
CGI-S		4.0 (0.1) [2-6]	
Duration of illness (years)		2.7 (2.3) [0.2-17]	
With over 5 years of illness		14 (17%)	
**Current medication**			
Aripiprazole		21	
Olanzapine		19	
Risperidone		7	
Paliperidone		5	
Lurasidone		2	
Flupentixol	None	1	
Zuclopenthixol		1	
Unmedicated		17	
Chlorpromazine daily equivalent doses (mg)		302.3 (19.3) [0-675]	

Values are expressed as number (and %) or mean (with standard error in brackets) [and range in square brackets], besides duration of illness that was expressed as median (and interquartile range). Abbreviations: BNSS = Brief Negative Symptoms Scale; CGI-S - Clinical Global Impression- Severity Scale; N = number of participants; PANSS = Positive and Negative Syndrome Scale; *t* = independent sample t-test; THC-positive UDS = delta-9-tetrahydrocannabinol positive urinary drug screen; χ = chi-squared test.

### Case–Control ROI QSM Analysis

**Figure 1:**
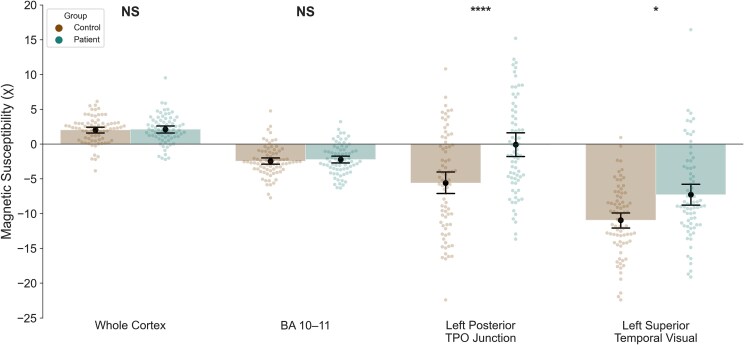
Group differences in magnetic susceptibility (χ) across selected cortical regions. Bars show group means with standard error, overlaid with individual participant values. Data from healthy controls are shown in brown; data from patients with schizophrenia are shown in green. In the predefined regions of interest, there were no significant differences in χ between groups for the whole cortex (*P* = .675) or Brodmann areas (BA) 10-11 (*P* = .537). In exploratory analyses, χ was significantly elevated in schizophrenia after false discovery rate correction in two left temporo-parieto-occipital (TPO) junction regions: the posterior third of the TPO junction (*P* < .0001) and the left superior temporal visual area (*P* = .033).

Figure 1 displays the results of the case–control analyses. There was no significant difference in whole cortex mean χ between healthy controls (mean = 1.49, SE = 0.21) and patients with schizophrenia (mean = 1.62, SE = 0.23; *d* = 0.07, 95% CI, −0.25 to 0.39, *P* = .675). Similarly, mean χ for the Brodmann areas 10-11 mask was not significantly different between groups in our primary analysis (mean = –2.44, SE = 0.25; mean = –2.23, SE = 0.25; *d* = 0.07, 95% CI, −0.25 to 0.39, *P* = .537).

Case–control results for the cortical ROIs are presented in Supplementary File 1. Two ROIs within the left temporo-parieto-occipital (TPO) junction remained significantly different after FDR correction, both showing elevated χ in schizophrenia. These were the TPO junction area 3, which comprises the posterior part of the TPO junction (*d* = 0.752, 95% CI, 0.419-1.084, FDR-corrected *P* < .0001), and the superior temporal visual area (*d* = 0.638, 95% CI, 0.308-0.967, FDR-corrected *P* = .034). Case–control difference in mean χ across these ROIs remained significant when controlling for potential clinical confounders and ROI cortical thickness (*t* = 3.01, *P* = .003; Table 2).

**Table 2:** Results from the Robust Linear Model Built to Predict Magnetic Susceptibility (χ) in the Two Left Temporo-Parieto-Occipital (TPO) Junction Regions of Interest (ROIs) with Higher χ in Schizophrenia than Controls with Case–Control Status, Potential Clinical Confounders, and ROI Cortical Thickness

Variable	Coefficient	Standard error	*t*-Score	*P*-value	[95% CI]	
(Intercept)	−8.28	6.38	−1.3	.194	−20.78	4.22
Schizophrenia group status	2.44	0.81	3.01	.003	0.85	4.03
Smoking history	2.06	0.97	2.13	.033	0.17	3.95
THC-positive UDS	−1.13	1.04	−1.09	.276	−3.17	0.91
Male sex	−0.92	0.9	−1.03	.304	−2.69	0.84
Age	0.05	0.06	0.77	.441	−0.07	0.17
ROI cortical thickness	−0.6	2.1	−0.28	.776	−4.71	3.52

### Correlating QSM in Significant ROIs with Clinical and Other Neuroimaging Measures

Table 3 shows the correlations between mean χ across the significant cortical ROIs with PANSS subscores, K_i_^cer^ in striatal subdivisions, χ in subcortical subregions, and SN-VTA NM-CNR. These cortical χ values were significantly negatively associated with K_i_^cer^ in the associative striatum (*r* = –0.37, FDR-corrected *P* = .048) and limbic striatum (*r* = –0.34, FDR-corrected *P* = .048), and SN-VTA NM-CNR (*r* = –0.35, *P* = .005).

**Table 3:** Correlations Between Mean Magnetic Susceptibility (χ) Across the Two Left Temporo-Parieto-Occipital (TPO) Junction Regions of Interest (ROIs), Which Showed Higher χ in Schizophrenia Compared with Controls, with Clinical and Neuroimaging Variables

Variable	*r*	*P*-value	FDR corrected *P*-value
**PANSS subscores**			
Positive	−0.11	.393	.590
Negative	0.02	.863	.863
General	−0.13	.327	.590
**Cortical thickness**			
Significant ROIs	−0.03	.798	N/A
**Striatal subdivision K**_**i**_^**cer**^			
Associative	−0.37	.021	.048
Sensorimotor	−0.18	.264	.264
Limbic	−0.34	.032	.048
**Subcortical subregion χ**			
Striatal	0	.969	.969
Thalamic	−0.26	.029	.118
Globus pallidus	0.16	.17	.339
SN-VTA	−0.02	.843	.969
**NM-CNR**			
SN-VTA	−0.35	.005	N/A

Abbreviations: K_i_^cer^ = dopamine synthesis capacity; NM-CNR = neuromelanin-sensitive MRI contrast-to-noise ratio; PANSS = Positive and Negative Syndrome Scale; SN-VTA = substantia nigra and ventral tegmental area; χ = magnetic susceptibility.

### Case–Control Voxelwise QSM Analysis

**Figure 2:**
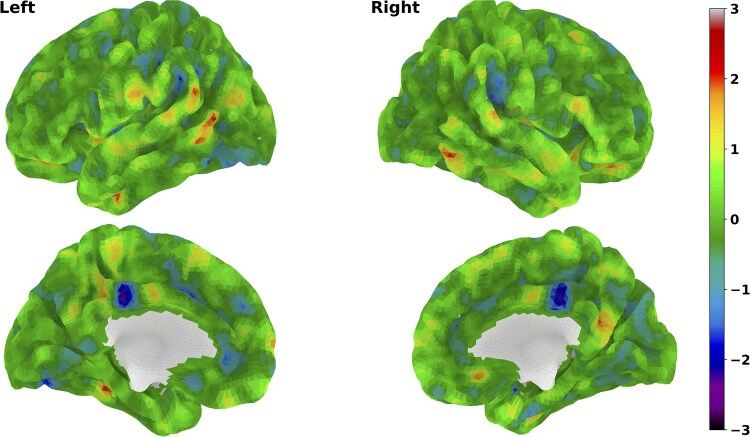
Surface-based statistical map showing voxelwise *t*-values for group differences in cortical magnetic susceptibility (χ). Positive *t*-values indicate higher χ in patients with schizophrenia than controls, and negative *t*-values indicate the inverse. The *t*-value color scale is shown on the right.

Figure 2 shows the surface-based t-values for cortical group differences in χ. Our voxelwise analysis identified a cluster within the left posterior middle temporal gyrus and TPO junction where schizophrenia was associated with greater χ (size = 1261 voxels, peak t-value = 5.62, FWE *P* < .05; Figure S3). Using the HCPex parcellations, approximately 36% of this cluster was in the posterior human temporal area (Area_PHT_L), 26% in the TPO junction area 3, and 23% in the superior temporal visual area. The remaining voxels were situated in other TPO junction ROIs. There were no significant clusters where χ was lower in schizophrenia.

## Discussion

Using QSM we present, to our knowledge, the first in vivo investigation of case–control cortical iron differences in schizophrenia. While prior postmortem work reported elevated iron in Brodmann areas 10-11 in schizophrenia,^4^ we found no case–control differences in mean χ for these regions or when examining the whole cortex. However, our exploratory analyses identified 2 ROIs in the left TPO junction with significantly higher χ in schizophrenia. Additionally, χ in these ROIs for the schizophrenia group inversely correlated with K_i_^cer^ in the associative and limbic striatal subdivisions and SN-VTA NM-CNR.

Notably, these results build upon previous findings from the same participant cohort, where patients with schizophrenia showed higher NM-CNR^36^ and lower χ in the SN-VTA.^9^ Both factors were independently linked to higher striatal K_i_^cer^ in the patient group. Collectively, our findings suggest that schizophrenia is characterized by regionally specific disruptions in brain iron homeostasis that contribute to mesostriatal dopamine dysfunction.

### Methodological Considerations

Accurately measuring cortical χ with QSM is technically challenging, particularly in areas prone to distortion, such as the orbitofrontal and inferior temporal cortices.^23^ We minimized the impact of distortion-related susceptibility artifacts by excluding voxels with extreme values and only including ROIs where a majority of voxels passed quality control. Dual masking with both GRE magnitude and T1w images increased our ability to remove artifact-prone areas. However, residual susceptibility artifacts likely reduced sensitivity in some cortical areas—particularly the orbitofrontal cortex—which may explain the absence of a case–control difference in Brodmann areas 10-11, despite postmortem evidence of increased iron here in schizophrenia.^4^

QSM measures the net magnetic susceptibility within a voxel by summing contributions from paramagnetic materials, which increase χ (eg, iron), and diamagnetic materials, which decrease χ (eg, myelin or calcium).^46^ Thus, higher cortical χ in schizophrenia may be explained by a loss of diamagnetic material. Case–control analyses measuring the T1w/T2w ratio, a proposed marker for intracortical myelin, have associated schizophrenia with elevated ratios in the left TPO junction, despite lower ratios in other cortical ROIs.^48^^,^^49^ As myelin accumulation should lower χ in schizophrenia, this is unlikely to explain the higher χ we observed. Furthermore, postmortem validation shows that T1w/T2w ratios correlate positively with χ, and even weakly negatively with myelin, suggesting that they are predominantly iron-sensitive.^50^ This is supported by the highest T1w/T2w ratios occurring in iron-rich, relatively myelin-poor subcortical regions.^50^^,^^51^ Thus, we interpret that higher χ and T1w/T2w ratios within the left TPO junction reflect iron accumulation rather than myelin loss. Nevertheless, we cannot exclude the possibility that cortical calcium loss, which has been reported in schizophrenia,^52^ could contribute to our QSM findings.

### Implications for Understanding Iron Homeostasis in Schizophrenia

Although peripheral iron deficiency is associated with schizophrenia, central and peripheral iron homeostasis are largely independent due to the blood–brain barrier.^5^ Findings from previous subcortical case–control iron-sensitive MRI studies have supported both high^31^^,^^53^ and low^7^^,^^8^^,^^54^ iron in schizophrenia. Our prior subcortical analysis of this participant cohort showed that subcortical χ was lower in schizophrenia.^10^ Therefore, our current finding that χ was increased in participants with schizophrenia within the left TPO junction but not in other cortical ROIs suggests that disease-related iron changes are regionally specific.

Elevated iron can drive neuroinflammation, oxidative stress, and lipid peroxidation, contributing to neurodegeneration.^5^ Although we did not identify a relationship between grey matter thickness and χ in the left TPO junction ROIs, cortical thinning^1^^,^^2^ and progressive grey matter loss^55^^,^^56^ are well-documented in schizophrenia. It therefore remains possible that focal iron accumulation may contribute to this pathology as expertly discussed by Lotan et al.^4^

Voxelwise analyses with structural MRI have linked grey matter loss in the left TPO junction to schizophrenia,^57^ particularly early-onset illness.^58^ Functional imaging studies report both hyperactivation and hypoactivation in the left temporoparietal subregion, with altered connectivity correlating with the severity of auditory hallucinations.^59–61^ Neuromodulation targeting this region, such as transcranial direct current stimulation, has shown benefits for negative symptoms and hallucinations in schizophrenia.^61^ Our finding of disrupted iron homeostasis in this area suggests a potential link between neurochemical and structural alterations, warranting further investigation.

The left TPO junction—which lies at the intersection of the temporal, parietal, and occipital lobes—is a hub for multisensory integration.^62^ It influences the striatum indirectly via projections to the prefrontal cortical network. In our schizophrenia group, higher χ in the left TPO junction significant ROIs was negatively correlated with K_i_^cer^ in the associative and limbic striatal subdivisions. This inverse correlation may be explained by disease heterogeneity as our sample likely contained a mix of patients with treatment-responsive and treatment-resistant schizophrenia. Treatment-resistance and chronic disease is associated with lower striatal K_i_^cer^^63^^,^^64^ and higher striatal χ^65^ than acute, treatment-responsive illness. Future studies in those with treatment-resistant illness are warranted to explore this hypothesis.

Our findings suggest that iron-related cortical dysfunction may impair cortical processing, altering corticostriatal glutamatergic signaling and contributing to striatal hyperdopaminergia. Microglia are a plausible contributor, as microglial activation increases iron uptake and storage,^66^ and postmortem studies indicate microglial dysfunction in schizophrenia.^67^ The additional inverse correlation of these χ values with SN-VTA NM-CNR suggests that this process may also influence midbrain dopamine function.

### Limitations and Future Directions

Although we applied FDR correction in our exploratory analysis, the risk of type I error remains. Replication in independent samples is needed to confirm whether χ is elevated in the left TPO junction in schizophrenia. Furthermore, applying FDR correction across 340 ROIs that passed quality control indicates a high risk of type II error. Performing this analysis on a larger dataset is necessary to examine whether case–control differences of a lower effect size exist for other ROIs.

Another limitation is that we did not observe significant associations between χ in the left TPO junction ROIs and PANSS symptom severity, which hinders the clinical interpretability of these findings. This null result may reflect the fact that most patients were receiving antipsychotic treatment, which is likely to reduce the variance in symptoms and consequently the power to detect relationships. We also lacked cognitive measures, which are relevant given that abnormal brain iron is implicated in both neurodegenerative cognitive disorders^68^ and cognitive development.^69^ Future studies in unmedicated first-episode cohorts are warranted to fully test the relationships between cortical χ with symptom and cognitive measures.

## Conclusion

In summary, we found localized increases in iron-sensitive QSM MRI signal within the left TPO junction in schizophrenia, which inversely correlated with striatal and SN-VTA dopamine synthesis capacity in patients with schizophrenia. This suggests that regional iron accumulation may contribute to cortical pathology and mesostriatal dopamine dysfunction in this disorder. These findings provide a foundation for further research into cortical iron as a potential biomarker and therapeutic target in schizophrenia.

## Supplementary Material

20251218_cortical_QSM_sup_sbag045

20251218_supplemental_file_1_sbag045
