# Supplementary material for: Cortical Iron and Mesostriatal Dopamine Function in Schizophrenia: A Positron Emission Tomography and Magnetic Resonance Imaging Study
Source: Schizophr Bull. 2026 Jul 23;52(4):sbag045. doi: 10.1093/schbul/sbag045 (PMC13391635; doi:10.1093/schbul/sbag045)
Supplement: 20251218_cortical_QSM_sup_sbag045 [file 20251218_cortical_qsm_sup_sbag045.docx]

**Supplementary Analyses**

**Figure S1.** Quantitative susceptibility mapping (QSM) images for a representative control participant and a patient. Magnetic susceptibility is shown in parts per billion (ppb).

**Figure S2.** Surface-based voxelwise mean QSM image in MNI space, derived from all participants whose scans passed quality control. Magnetic susceptibility values (χ) are shown in parts per billion (ppb).

**Figure S3.** Voxelwise cluster within the left posterior middle temporal gyrus and temporo-parieto-occipital junction showing significantly greater magnetic susceptibility (χ) in patients with schizophrenia compared to matched healthy controls. The cluster (shown in red) comprised 1261 voxels, with the peak voxel (t-value = 5.62) at Montreal Neuroimaging Institute coordinates x = –54, y = –73, z = 20. This result survived family-wise error correction (p < 0.05).
